# Measuring happiness under interpersonal comparison: An advanced theoretical framework and implications

**DOI:** 10.1371/journal.pone.0261407

**Published:** 2021-12-16

**Authors:** Junyi Chai

**Affiliations:** 1 Division of Business and Management, BNU-HKBU United International College, Zhuhai, China; 2 Centre for Evaluation Studies, Beijing Normal University (Zhuhai Campus), Zhuhai, China; 3 Department of Management and Marketing, The Hong Kong Polytechnic University, Hung Hom, Kowloon, Hong Kong, China; The Open University, UNITED KINGDOM

## Abstract

The origin of happiness arouses people’s curiosity for a long time. Recent research introduces a utility theory for measuring subjective happiness in a social context. The past recent monetary conditions influence the present subjective happiness through two distinct channels: interpersonal comparison and self-adaptation. In this paper, we develop this theory to analyze behavioral patterns. Together with prospect theory’s gain-loss utility function, we exploit the theory in predicting psychological phenomena of craving. We explore the relationships between happiness and earnings. Under certain conditions, a high payoff disappoints you immediately and even leads to continuous disappointment across periods. We extend the explanations of the scenarios of New York cabdrivers’ labor-supply decisions. The effect of social comparisons may trigger workers’ behaviors of quit-working, which deepen related understandings of the literature.

                              *"To be happy, we must not be too concerned with others."*

                                        –*Albert Camus in < La Chute > (1913–1960)*

## 1. Introduction

Whether money buys happiness has been frequently asked questions in both casual communications and academic debates. Falk and Graeber [1] uncover that people’s prosocial behavior produces a delayed negative effect on happiness through the experiments of saving a human life. More recently, the literature [2] developed a model of moment utility with three state variables: retaliation, aspiration, and ambition. The retaliation reduces moment utility due to social comparison. The aspiration reduces utility due to people’s self-adaption to the past consumption of people per se. The ambition plays like a platform that comes from recent consumptions under social comparisons. The total utility measures people’s happiness on their past and current payoffs in a social context.

Using the utility in measuring happiness is not new. Bentham in 1789 [3] could be the first to use the utility notion to represent people’s pleasure or pain experienced. Two assumptions exist in his conceptualization of happiness: (1) goodness and badness of experience are quantifiable and (2) the quantities obtained can be added across people. Pareto (1848–1923) rejected this idea of discrete utilities and considered a pairwise comparison. The utility function in Pareto’s idea reflects human preference and nothing more. Kahneman et al. [4] distinguished Bentham’s concept of utility; forcefully advocated the Experienced utility (EU) in measuring happiness that relies on revealed preference as Decision utility (DU). They further distinguished the EU from DU of economics. Kahneman [5] argued, “An explicit distinction between two notions of utility. The experienced utility of an outcome is the measure of the hedonic experience of that outcome…. The decision utility of an outcome, as in modern usage, is the weight assigned to that outcome in a decision”. Earlier studies on the EU framework can be found in [6–8]. Using EU for measuring subjective happiness of individuals is prevailing, such as the literature [2, 9, 10].

Chai’s theory [2] accommodates both extrinsic and intrinsic determinants of subjective happiness. Interpersonal comparison is an extrinsic determinant captured by the ambition factor. Self-adaption is an intrinsic determinant captured by the aspiration level. The wellbeing (WB, henceforth) model captures both determinants. Its degenerated models such as the ambition (AM) model and the aspiration (AS) model can independently capture extrinsic and intrinsic determinants. Several unique features exist. It distinguishes satisfaction from desirability; and, further distinguishes happiness from satisfaction. This is quite compelling as it explains the scenarios like the happy poor and the sad rich in Zapf’s welfare positions [11]. Such distinctions are often omitted before [12]. Closed to the problem raised in our paper, the literature [13] studied how the sudden acquisition of a large sum of money produces negative consequences and causes greater unhappiness at the individual level. Interestingly, they distinguished between the pecuniary and non-pecuniary parts of individual happiness, whereas Chai’s theory [2] distinguishes one’s happiness that comes from the comparisons with himself and his competitors.

In this study, the terms wellbeing and happiness can be used interchangeably in many cases. A slight difference is that happiness majorly pertains to a period, whereas wellbeing pertains to a time span that contains several periods. Henceforth, we call the hybrid model as the wellbeing (WB) model. We call the AM and AS models with the consistency of [2]. All these models are called the happiness models.

Although the theory of [2] is state-of-the-art for measuring happiness at the micro level, it leaves room for further utility specifications. In this paper, we extend Chai’s general theory of happiness [2] to an advanced theoretical framework by imposing the prevailing prospect theory’s (PT henceforth) "gain-loss" (GL) utility as the specifications of the utility. The GL utility formula is "S-shaped" and separated as the gain part and the loss part. The functional curve is concave for gain and convex for loss. This utility exhibits loss aversion under which people are more bothered by losses than by gains. It combines the features of diminishing sensitivity and preference dependence. Underpinned by these fundamental features of behaviors, our advanced theoretical framework has attractive properties and great potential for implications and applications. Specifically, we exploit three aspects of implications and applications of our advanced theory, including:

1. The predictions in the psychological phenomenon of craving and its applications (Section 3).
2. The predictions in the influence of "wealth shock" on individual happiness (Section 4).
3. The prediction in self-determined workers’ labor-supply decisions (Section 5).

As the first prediction, we explore the model in capturing a psychological phenomenon—craving. People experience craving if moment utility over a unit payoff is higher than the utility at the neutral level. In craving status, a normal level of wage brings an abnormally high level of happiness. The condition of producing craving is the existence of a negative ambition level. In AM, craving is determined by the retaliation. In WB, craving is determined by the combination of retaliation and aspiration.

As the second prediction, we explore the WB model in predicting relationships between happiness and earnings. Intuitively, a high payoff is supposed to bring a high level of happiness. However, we found that an exceptionally high payoff may surprisingly result in a disappointment immediately and even continuous disappointments across periods. We explore the mechanisms and conditions of disappointment from high-grade pay, where the endogenous compassion factor and the endogenous speed of establishing aspiration pay determinant roles. Therefore, more money cannot necessarily buy you more happiness and continuously disappoint you if certain endogenous conditions are met.

As the third prediction, we explore the relationship between happiness and willingness to work in a scenario of labor-supply decisions. Considering workers seek happiness and avoid disappointment, an abnormal high pay in this hour may disappoint them immediately and further trigger their unwillingness to work afterward. The determinants are people’s endogenous characteristics: the compassion factor and the speed of establishing aspiration. Unlike past theoretical explanations of this well-studied scenario in literature, we uncover the effect of social comparisons on such time-flexible self-determined workers’ labor-supply decisions.

The rest of this paper is structured as below. Section 2 presents the theoretical framework of the models by assuming the PT’s GL utility and discusses the EU’s usages in measuring happiness. Section 3 studies how the model captures craving. In Section 4, we study the influence of happiness from the sudden acquisition of wealth. Section 5 studies the influence of happiness on workers’ labor-supply decisions. Section 6 concludes this paper and outlines the directions for future works.

## 2. The advanced theoretical framework

### 2.1. The happiness model under gain-loss utility

The general theory of [2] refers to the WB model that is a hybridization of the AM model and the AS model. The ambition notion is more closed to a psychological measurement thereby it relies on interpersonal differences and is positively related to attainment [14]. An ambition level is a platform of consumption in which past internal and external factors influence the moment utility. The aspiration notion resembles an economic concept of habit formation [15] whereby the past accumulated level reduces the moment utility. The retaliation notion roots in the insight of Fehr and Schmidt’s fairness model [16] thereby people dislike being behind more than they dislike being ahead in interpersonal comparisons. As Kahneman and Thaler [8] wrote, “a richer and more psychologically realistic characterization of the quality of experiences that includes the following additional factors: adaptation, contrast, interpersonal comparison, loss aversion, and fairness.”

However, the functional formulas in the original WB, AM, and AS models are not specified as a general framework. In this paper, we advance this general framework by further assuming a universal S-shaped utility that is exactly a PT’s utility function. The utility formula entails (a) the concavity for the positive zone (gains), (b) the convexity for the negative zone (losses), (c) a steeper loss than gain limb, and (d) certain forms of loss aversion [17]. The advanced model presented in this paper inherits all properties of Chai’s general model [2] and further possesses the great potential of implications and predictions studied in Sections 3–5. Without a particular declaration, the WB model assumes the GL utility is used for all present results.

The WB model formally measures an individual A (her)’s happiness influenced by relative payoff comparisons interpersonally. If the A considers her peer B as a competitor, the characterized ambition level *S* formulates how the context of the last period influences her subjective happiness at present. The moment utility replaces *u*(*x*) by *u*(*S*+*x*−*R*−*P*)−*u*(S). The total utility can be measured as below.

$$V(x_{1},\ldots ,x_{T})=\sum_{t=1}^{T}\left[u(S_{t}-R_{t}+x_{t}-P_{t})-u(S_{t})\right]$$

where three state variables for *t* = 2,…,*T*:

1. The ambition: *S*_*t*_ = *β*(*S*_*t*−1_−*R*_*t*−1_+*x*_*t*−1_−*P*_*t*−1_), for *β*∈[0,1] and the given *S*_1_;
2. The aspiration: *R*_*t*_ = *R*_*t*−1_+*α*(*x*_*t*−1_−*R*_*t*−1_) for *α*∈[0,1] and the given *R*_1_;
3. The retaliation: *P*_*t*_ = *λ* max{*y*_*t*_−*x*_*t*_, 0}+*μ* max(*x*_*t*_−*y*_*t*_, 0}, for *μ*∈[0,1) and λ≥μ.

The value of *S*_*t*_ indicates the A’s ambition level at period t that is established in past circumstances. The ambition retention factor *β* parameterizes the speed of establishing such an ambition level. It captures the carryover effect across periods, which is the extent of retaining the period utility level from the past to the present. The carrier of moment utility is the increment from the current ambition level rather than the current, actual payoff. At each period, the present ambition is a benchmark of measuring her perceived happiness at that period over the actual payoff (*x*_*t*_). Unless stated otherwise, we set *S*_1_ = 0.

The value of *R*_*t*_ measures the A’s self-adaptation at period t through accumulating past payoffs. The theory in [2] imposes the retaliation to exhibit individuals’ habituation and thus uses an exponential smoothing of past payoffs after assuming adjacent substitutability [18]. If considering *R*_*t*_ rather than *S*_*t*_, the WB model is exactly a habituation model where the moment utility *u*(*x*_*t*_−*R*_*t*_). The intrinsic variable may not be unique; for example, a model of stronger habituation, namely addiction, is also legitimate in the theoretical framework. In this paper, we maintain the original formulation of aspiration and may discuss possible variations in due course.

The value of *P*_*t*_ measures how social comparisons enter the utility formula. Retaliation models fairness thinking under which people are self-centered inequity aversion [16]. This state variable is an additive combination of what we call an envy component and a compassion component. The envy presents *λ* max{*y*_*t*_−*x*_*t*_, 0} for the envy factor *λ*, while the compassion presents *μ* max{*x*_*t*_−*y*_*t*_, 0} for the compassion factor *μ*. The retaliation *P*_*t*_ is exactly the moment envy-compassion level at the period t, in which people dislike being behind more than they dislike being ahead of their peers. In the literature, fairness thinking is not the unique mode in social comparisons. Long and Nasiry (2020) assume people have ahead-seeking preferences in which the transparency on employee’s payments could more benefit to the firm. The basic notation is summarized in Table 1.

**Table 1:** Notation for setup and preference parameters in the WB model.

*V*(*x*_1_,…,*x*_*T*_)	Total utility, given by $\sum_{t=1}^{T}\left[u(S_{t}-R_{t}+x_{t}-P_{t})-u(S_{t})\right]$	Utility
*u*(∙)	Moment (per-period) utility	
*x*_*t*_	The actual payoff at the period t	Payoffs
*y*_*t*_	The peer’s actual payoff at the period t	
*S*_*t*_	Ambition level; given by *β*(*S*_*t*−1_−*R*_*t*−1_+*x*_*t*−1_−*P*_*t*−1_)	State Variables
*R*_*t*_	Aspiration level; given by *R*_*t*−1_+*α*(*x*_*t*−1_−*R*_*t*−1_)	
*P*_*t*_	Retaliation level; given by *λ* max{*y*_*t*_−*x*_*t*_, 0}+*μ* max{*x*_*t*_−*y*_*t*_, 0}	
*μ*	Compassion factor (aversion to being ahead), *μ*∈[0,1)	Intrinsic Parameters (all given)
*λ*	Envy factor (aversion to being behind), *λ*≥*μ* for *μ*∈[0,1)	
*α*	Speed of establishing aspiration: *α*∈[0,1]	
*β*	Speed of establishing ambition: *β*∈[0,1]	

The basic assumption of our model is the fact that people’s subjective happiness getting from consumption is affected by the consumption levels of other people. This is intuitive and rarely disputed in economists and psychologists. The envy and compassion factors are key parameters to capture peers’ (external) influence on the internal value of subjective happiness. Banerjee [19] could be one of the first to analyze envy as a particular externality of happiness. Followed by Boiney [20], individual envy is modeled under a cardinal von Neumann-Morgenstern utility function through pairwise comparisons interpersonally. Interestingly, envy and its counterpart—compassion—enter our model through interpersonal comparisons as well, but different in a particular form of the state variable what we have called the retaliation level Pt.

**Fig 1:**
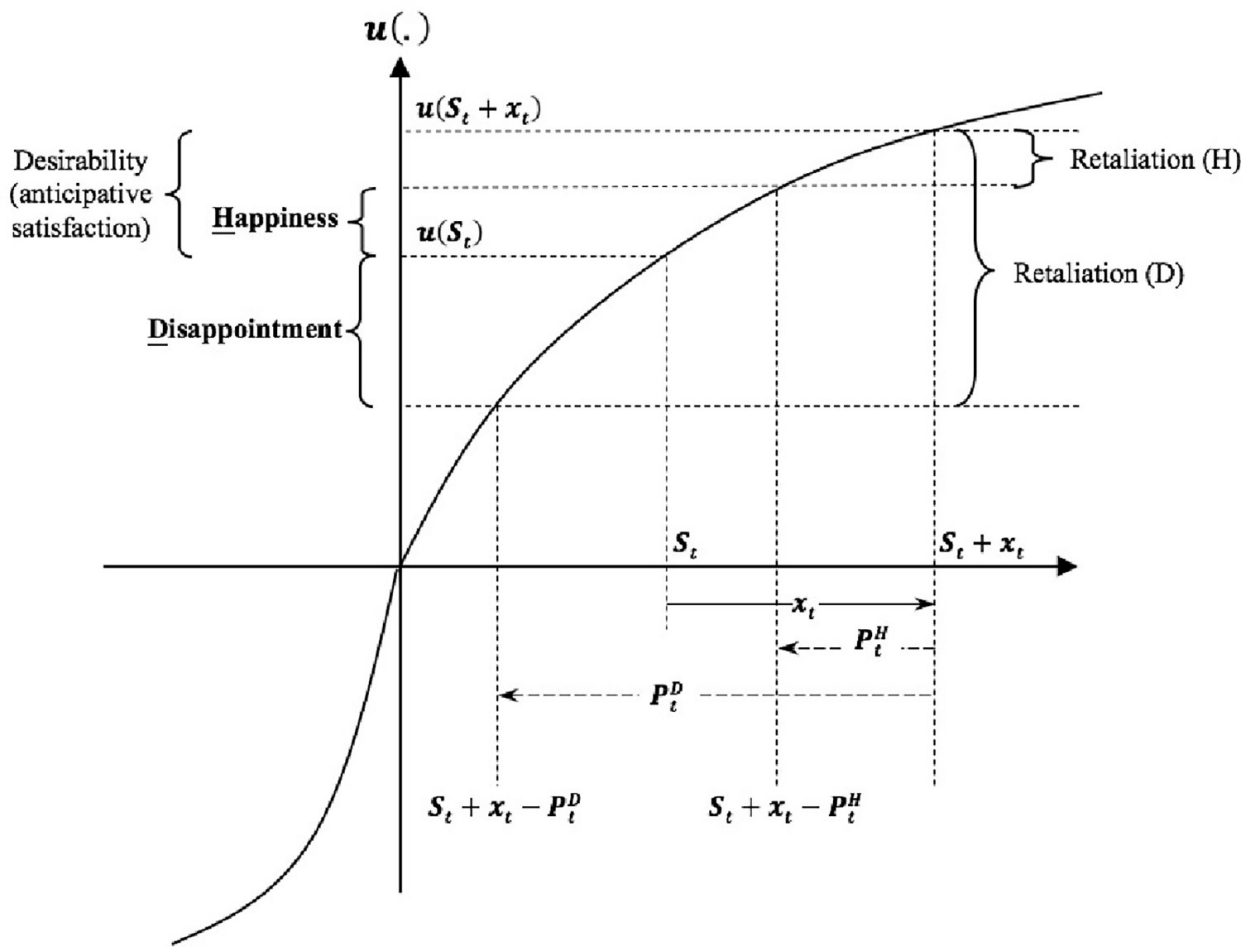
State variables of AM under the S-shaped utility.

The noteworthy degeneration of the WB model is the AM model after excluding the intrinsic aspiration factor. The AM carries most of the properties of WB. If further waiving influences of social comparisons under AM, the happiness degenerates as the satisfaction (satiation) measured as u(S_t_+x_t_)−u(S_t_), which resembles a satiation model of Baucells and Sarin [9]. If the state variable *P*_*t*_ is not waived, the utility of retaliation is u(S_t_+x_t_)−u(S_t_+x_t_−P_t_). Experiencing happiness is conditioned by her satisfaction exceeds her retaliation; otherwise, people experience a disappointment measured by u(S_t_)−u(S_t_+x_t_−P_t_). The disappointment can be interpreted as a reference-dependent "unhappiness" where P_t_ pertains a kind of reference point of x_t_. Thus, the model incorporates a neutral state—"zero utility", under which people are neither happy nor unhappy. Fig 1 illustrates state variables of AM under the S-shaped utility.

These state variables in the WB model have the same implications as those in the AM model but become more complicated after the aspiration level is entered. The net effect of R and S is the adjustment level given by *Δ* = *S*−*R*. The desirability is the marginal utility of wage *x* as given by *u*(*Δ*+*x*)−*u*(*Δ*). Retraction distinguishes (actual) satisfaction from desirability (i.e., anticipated satisfaction); is measured by *u*(*S*)−*u*(*Δ*). Frustration distinguishes happiness from satisfaction; is measured by *u*(*Δ*+*x*)−*u*(*Δ*+*x*−*P*). There is no frustration in AS and no retraction in AM. Both AS and AM models are degenerations of the WB model. In Table 2, we summarized all state variables of the AM, AS, and WB models.

**Table 2:** Measurements in the AM, AS, and WB models.

	The WB Model	The AM Model	The AS Model
	(S≠0, R≠0, and *Δ* = *S*−*R*)	(R = 0)	(P = S = 0)
Frustration	u(*Δ*+x)−u(*Δ*+x−P)	u(S+x) −u(S+x−P)	N/A
Retraction	u(S)−u(*Δ*)	N/A	−u(−R)
Disappointment	u(S)−u(*Δ*+x−P)	u(S)−u(S+x−P)	−u(x−R)
Desirability	u(*Δ*+x)−u(*Δ*)	u(S+x)−u(S)	u(x−R)−u(−R)
Satisfaction	u(*Δ*+x)−u(S)	u(x−R)	u(S+x)−u(S)
Happiness	u(*Δ*+x−P)−u(S)	u(S+x−P)−u(S)	u(x−R)

In our advanced theoretical framework, (a) EU measures hedonic and affective experience that is cardinal but different from DU; (b) the total utility across periods is the sum of per-period EU; and (c) EU depends on past outcomes or past and current experiences, or cultural and social influences. EU can be based on the moment (instant utility) or the memory (remembered utility), which are interpersonally comparable and share a common scale by people [4] (p. 383). The per-period utility is based on individual experience (feelings of pleasure or pain) over outcomes (payoffs), rather than revealed actual choices. Therefore, using the EU in a riskless environment is legitimate in measuring cardinal happiness, as [21] (p. 405) emphasized. This principle also applies to the original theory of [2]. We further discuss this principle in Section 2.2.

### 2.2. Measuring happiness in experienced utility

The behavioral phenomenon is assumed to correspond to people’s mental states. It is important to distinguish different varieties of utility, which is also valuable when using neuroscience tools to be interpreted [22]. Following [4], we understand utility from three perspectives: (1) DU—the weight of potential outcomes in decisions; (2) EU—the original concepts of utility from Bentham, focusing on the instant experience of pleasure and pain; (3) anticipation utility (mainly in psychologists’ view)—highlighting the importance of utility related to anticipating a positive or negative outcome [23]. A major determinant of the EU is “remembered utility”. The literature [24] gives a typical example of it: people may decide to have a cigarette (subject to DU), yet be better off if they don’t have it (subject to EU). The DU is determined by choices, whereas the EU is determined by the psychophysical method. The key problem is how to measure the goods through utility. In Benthamite, the criterion is whether it maximizes pleasure or minimizes pain. EU meets this criterion, but DU is not. “A separate value judgment” in [4] is necessary before Bengthamite utility can be identified with the good.

The DU relies on the actual choices that is more suitable for object-oriented analyses and judgments, but fails to measure “personal utility situation”. When facing a few alternatives with several evaluation criteria, DU is usually used to measure each alternative for ranking or sorting alternatives. Unlike the DU is objective measurements, the EU tends to be psychological measurements. Read [24] further specified a strong version and a weak version of the EU, where the strong version is more closed to Bentham’s idea of utility. Maximizing personal EU is not the utilitarian principle, which also entails social welfare. For the strong version, the key point is finding “an objective index of the good”, where the goods can be made objectively better or worse. The candidate index can be measured or assessed, and the index thus measured must be goods, like durable, non-durable, or memorable goods [25].

The predictive utility is a belief about the future experience, which does not influence the current EU. The t-period payoff event produces an instant utility. Standing on t, the past payoffs produce remembered utility to the present. Meanwhile, the future scheduled payoffs produce predictive utility to the present. The t-period happiness is influenced by remembered and instant utility but not influenced by the predictive utility. If our target is to figure out how the future scheduled payoffs influence the entire wellbeing over a scheduled payment scheme, the predictive utility and the present bias become crucial. For example, there exists a fixed period whereby people receive payoffs regularly; a three-year monthly-pay contract pays a junior faculty of a university. Kahneman and Tversky [26] (Chapter 42) stated, “People […] may lack skills in predicting their future tastes and can be led to erroneous choices by fallible memory and incorrect evaluation of past experiences.” Past payoffs influence current experiences of happiness through the channels of aspiration and ambition, both of which are subjective retrospective reports of pleasure or pain derived from interpersonal comparison and self-adaptation. In the WB model, the speed of establishing aspiration and ambition (the coefficients α and β, respectively) can be interpreted as formulating the bias of remembered utility.

## 3. Happiness from craving

Craving as a psychological phenomenon could be triggered by interpersonal comparison. Loewenstein [27] argued that people often behave against their self-interest due to the feeling of being “out of control”, which come from a visceral could influence such as craving. Goldstein [28] says that craving is an intense and overwhelming desire that leads to impulsive and vague decisions. Baucells and Sarin [10] formulated that craving comes from negative satiation on past consumptions that further derives from the unmet need in the past. This section studies the mechanisms and implications of craving predicted by the AM model (Section 3.1) and the hybrid WB model (Section 3.2).

### 3.1. Craving in the AM model

We begin by considering a two-period setting under the framework of the AM model. Assume that the per-period utility function *u* is S-shaped and exhibits certain loss aversion. Given a payoff stream (x_0_, x_1_), the moment happiness represents *u*(*S*_0_+*x*_0_−*P*_0_)−*u*(*S*_0_) at *t* = 0 and *u*(*S*_1_+*x*_1_−*P*_1_)−*u*(*S*_1_) at *t* = 1. Given *S*_0_ = 0, the moment happiness represents *u*(*x*_0_−*P*_0_) at *t* = 0 and the ambition accumulates up to *S*_1_ = *β*(*x*_0_−*P*_0_).

Three situations can be distinguished here. First, an individual A is at a neutral status if *x*_0_ = *P*_0_, where for *t* = 1 the ambition cannot be created as *S*_1_ = 0. Second, the A experiences happiness if *x*_0_>*P*_0_, where for *t* = 1, the ambition is accumulated and positive as *S*_1_>0. Third, the A experiences disappointment if *x*_0_<*P*_0_, where the ambition is accumulated and negative as *S*_1_<0.

**Fig 2:**
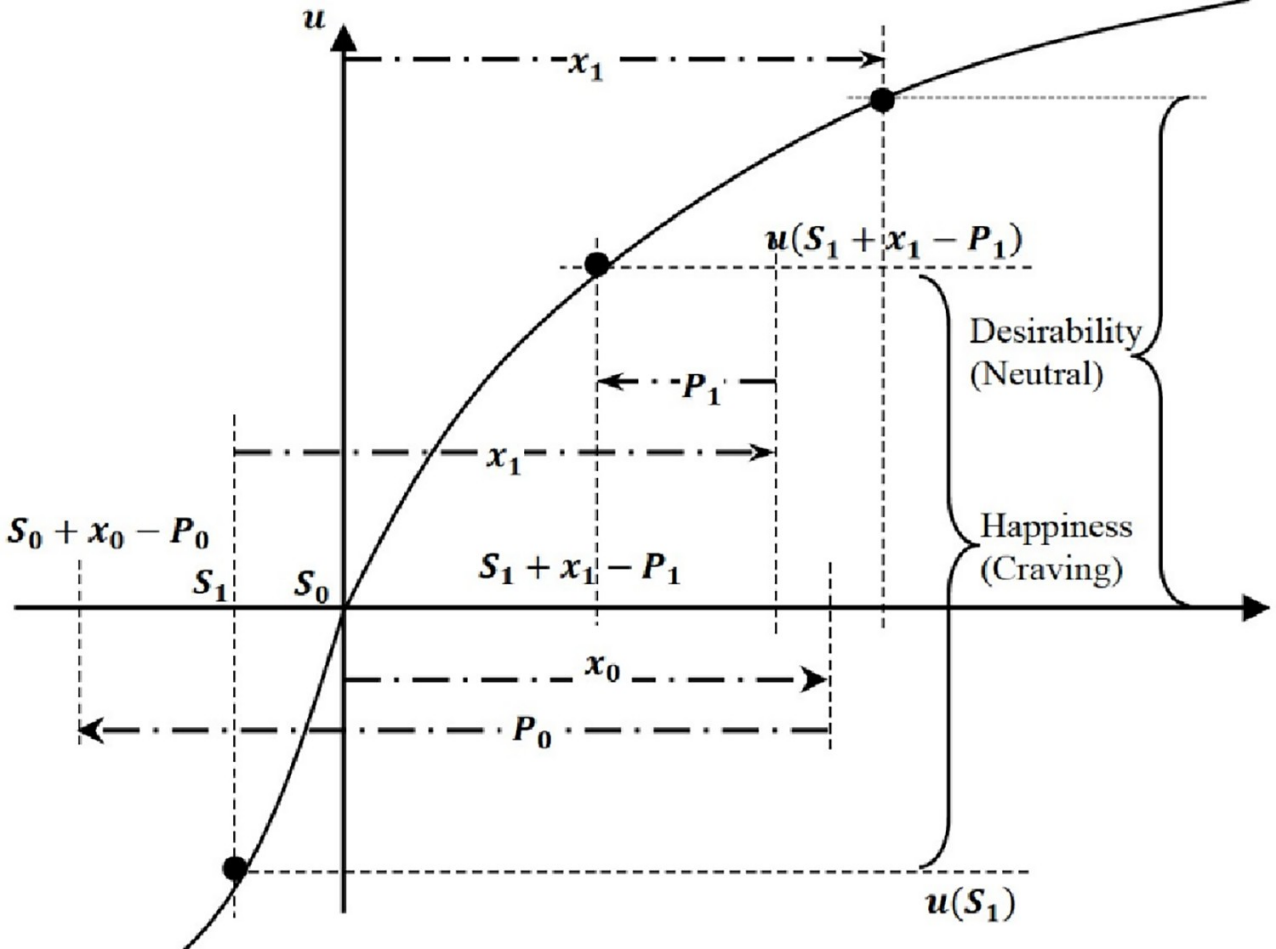
Mechanisms of craving in the AM model.

With all this in mind, the third situation (i.e., *S*_1_<0) exists a possibility that the moment utility over *x*_1_ is higher than the desirability over *x*_1_ at the neutral level. The zone between *S*_1_ and *S*_1_+*x*_1_−*P*_1_ in the horizontal axis maps to the steepest curve of the utility function around the kink point, as given a concave curve for gain and a convex curve for loss. That is, the moment happiness will be greatly amplified once a negative ambition level is accumulated in the previous stage. Fig 2 illustrates this process.

After grasping craving by intuition, now we formally define craving below. People experience craving if *u*(S+1−P)−*u*(S)>*u*(1): the moment utility over a unit payoff is higher than that at the neutral level. The inequality says that happiness over a payoff in social comparisons is surprisingly higher than happiness without considering the social context. The moment utility under craving can be greatly amplified due to the existence of interpersonal comparisons. There exists an intermediate status when *u*(S+*x*−P)−*u*(S)>*u*(*x*−P). It says that historical contexts amplify the moment utility over a retaliation-adjusted payoff, denoted as *x*−P, that is the net outcome of the present situation. Our definition of craving provides a stronger condition than this intermediate status.

Modeling craving implies that social comparisons are not always going against creating subjective happiness. In our theoretical framework, the retaliation that characterizing interpersonal comparisons is a negative correlation with moment happiness. It works for most "normal" cases in which the ambition is accumulated and positive. However, under certain abnormal conditions that hold a negative level of ambition before, the craving will be triggered and further greatly amplify the level of happiness in the subsequent period. In the following proposition, we present the condition for normal cases that avoids craving.

**Proposition 3–1.** Under the GL utility *u*, craving can be avoided if any of the following conditions are satisfied:

(A) *x*_*t*_≥*y*_*t*_; (B) *x*_*t*_<*y*_*t*_ and *x*_*t*+1_/*y*_*t*+1_≤*λ*/(*λ*+1) for *λ*∈[0, ∞),

Proof of Proposition 3–1. (A) Craving happens when *S*_*t*+1_<0. Suppose *x*_*t*_≥*y*_*t*_, we have *P*_*t*_ = *μ*(*x*_*t*_−*y*_*t*_)≤*x*_*t*_ for *μ*∈[0,1) that contradicts the condition *S*_*t*+1_ = *β*(*x*_*t*_−*P*_*t*_)<0. That is, an individual never holds a negative ambition at t+1 if her payoff exceeds her peer’s payoff at period t. Being behind in social comparisons at period t is a necessary condition for craving at period t+1. (B) At period t+1, the retaliation influences moment utility independently. Producing craving entails *x*_*t*+1_>*P*_*t*+1_. We hold *x*>*P* if *x*>*y* at t+1. If *x*<*y*, however, there exists *x*≤*P* = *λ*(y−x) that leads to *x*≤*P*. Therefore, the individual never experience craving if (1+*λ*)*x*≤*λy* at the period t+1 for *λ*∈[0, ∞).

Interpersonal comparisons could solely trigger the craving for money under certain conditions. At any period, A’s disadvantageous status could produce a negative ambition level that arises a craving status. Regains of a normal level of payoffs are likely to produce a higher level of happiness than that at a neutral status. Craving comes from the disadvantageous status in relative payoff comparisons interpersonally, the psychological reaction of aversion to being behind, and the negative ambition level that comes from the past situations of interpersonal comparison. The implications of the AM model have enriched the state-of-the-art understandings on craving among the literature.

### 3.2. Craving in the WB model

Under the framework of the WB model, people will experience craving if *u*(S−R+x−P)−*u*(S)>*u*(x) is satisfied with S<0. Compared with AM, the aspiration level R produces an additional influence on craving. We first consider a retaliation-adjusted payoff denoted as xp = x−P at all periods. Given $x_{t}^{0}=0$, we hold $xP_{t}=x_{t}^{0}-P_{t}=-\lambda y_{t}$ for *λ*∈[0, ∞). At the period t, the individual A surely experiences disappointment denoted as DIS(t) = *u*(*S*_*t*_)−*u*(*S*_*t*_−*R*_*t*_−*λy*_*t*_). Based on the mechanisms of WB, DIS(t) is subject to the "benchmark" ambition *S*_*t*_ and the relative value between *S*_*t*_ and *R*_*t*_+*λy*_*t*_.

Three situations could produce more disappointment, including (i) a higher *R*_*t*_, (ii) a higher *λ*, and (iii) a higher *y*_*t*_. For (i), if *R*_*t*_ is large enough until exceeding *S*_*t*_, we derive *S*_*t*_<*R*_*t*_+*λy*_*t*_ since *λy*_*t*_≥0. When *S*_*t*_>*R*_*t*_, we then consider the situations (ii) and (iii). A higher *λ* and a higher *y*_*t*_ lead to *S*_*t*_<*R*_*t*_+*λy*_*t*_, respectively. The inequality further implies a negative ambition level at t+1, because of *β*(*S*_*t*_−*R*_*t*_−*λy*_*t*_) = *S*_*t*+1_<0. Assuming the GL utility, a higher *R*_*t*_ or *λ* or *y*_*t*_ produces a higher DIS(t) at the period t and produces a lower and negative *S*_*t*+1_ at the period t+1. A negative ambition level possibly triggers a craving.

The moment happiness at period t+1 presents HAP(*t*) = *u*(*S*_*t*+1_−*R*_*t*+1_+*xp*_*t*+1_)−*u*(*S*_*t*+1_). Aspiration is shrunk as *R*_*t*+1_ = (1−*α*)*R*_*t*_. Conditioned by a negative ambition level at t+1, craving is triggered if HAP(*t*)>*u*(*x*_*t*+1_). The amplified HAP(*t*) derives from the property of the GL utility −*u*(−*x*)≥*λu*(*x*) for *x*>0 and *λ*>1—the steep curve in the positive zone and the steeper curve in the negative zone around the kink point. Therefore, craving in WB carries the properties of carving in AM. Yet, unlike AM, the cause of a negative ambition can be two-fold: the accumulated aspiration and the non-accumulated retaliation.

To sum up, a negative ambition is a necessary but insufficient condition for craving under the framework of WB. A high *λ*, *y*_*t*_, or *R*_*t*_ can produce a negative *S*_*t*+1_, independently or unitedly. A person, who has accumulated a high aspiration or tends to envy others (i.e., the strong aversion to being behind), is more likely to experience craving. Comparisons with the peer who has a higher payoff motivate people to build a negative level of ambition. Resuming a payoff fulfills the previously unmet need, which produces amplified happiness than the happiness at the neutral status.

Breiter et al. [29] advocate that their actual payoffs and unmet payoffs can influence people’s happiness. Craving can be interpreted as a mental reaction of disappointment on previous payoffs: people tend psychological compensation across periods. A busy-working father could be willing to compensate his daughter by spending more time with her. A hungry man tends to overeat once he has a rich meal. A poor man tends to have overconsumption once s/he becomes rich suddenly. Such compensation mentalities are universal.

An individual is at a craving status if HAP(t)>*u*(*x*_*t*_) conditioned by *S*_*t*_<0. The nonpayment in the past is a special case for arising cravings. The inequality *S*−*R*+*x*−*P*<0 at *t*−1 is a necessary but insufficient condition of craving at period *t*. This condition can also present as x<(λy−Δ)/(λ+1) for y>x, λ≥0, and Δ = S−R. The nonspecial case in the WB model should be S<0<R. WB degenerates AM when S<0 = R. WB degenerates AS when S = 0≤R. There is no craving in AS because ambition is not involved. Over *t*, we further analyze the special cases that involve different situations between S and R as below.

- When R = S = 0, the long-term absence of payoffs makes S and R at the period t shrink to zero. Regaining a payoff at t will not generate a craving, yet it can be deemed as the beginning of a new payoff stream. If the t-period retaliation P exceeds the regained payoff *x*, however, it leads S_t+1_<0, which further possibly generate craving at t+1. In both AM and WB, craving is caused by interpersonal comparison, which depends on the circumstance of the last period rather than the past payment stream.
- When R>S>0, individual A holds a positive ambition and a higher aspiration. Retraction is thus very large since it covers a portion of the steepest utility curve around the kink point. It is easy to prove that this case cannot produce craving at period t. On the one hand, u(S−R+x)<u(x) is valid for S<R. On the other hand, u(S−R+x)≥u(S−R+x−P)>u(S−R+x−P)−u(S) is valid for *S*<0. Thus, we hold u(S−R+x−P)−u(S)<u(x) that contradicts our definition of craving.

### 3.3. A discussion

Under certain conditions, [10]’s satiation and habituation (SH) model has similar predictions on craving. Our definition of craving resembles that of [10]: the moment utility over a unit outcome is higher than that at the neutral level. Under the GL utility, craving is caused by a negative benchmark of calculation that can be the satiation level in [10] and the ambition level in the AM/WB model. However, fundamental differences exist. First, craving in the SH model is caused by stopping consumptions on a habitual good; the negative satiation level is triggered by habituation accumulated before. But craving in AM is caused by interpersonal comparisons in past periods; craving in WB is caused by interpersonal comparison and self-adaptation unitedly. Second, craving in the SH model is due to the accumulation of non-consumptions. But craving in AM/WB is noncumulative over time. A negative ambition level can be built by just one past period rather than a payoff stream in the past. Thus, craving in AM/WB could happen in any period only if an overwhelming level of retaliation exists in the last period. Third, craving in the SH model is subject to past situations, whereas craving in AM/WB is subject to the current social context in addition to past situations.

Note that the outcomes of utility in AM/WB must be comparable interpersonally. Some habit-forming goods modeled by SH may not become the outcomes in AM or WB, for example, drugs, alcohol, or food. It is difficult to say that a wonderful dinner experienced by your friends (peers) would be able to influence your happiness for having dinner. Your feeling (happy or disappointing) of drinking a beer is relatively independent of your peers’ perception of drinking their beer. Consumptions of foods or a beer can be well formulated by satiation or habit formation as the SH model does. Yet, they are not suitable for AM or WB since lacking social comparability. Although moment utility in SH has been considered EU and a measure of happiness, conclusively, differences between SH and AM/WB are significant and fundamental.

Chai [2] forcefully argued that some things like sex and health are lack of interpersonal comparability. As existing certain internal standards, these things can be well modeled by individual satiation and habit formation (e.g.,[10]’s SH model). Yet, as mentioned in [2] (footnote 4), these things may become socially comparable under certain cultures or backgrounds. For example, alcohol drinking in Korea, Russia, or northern China could represent certain interpersonally comparable capacities thereby the WB and AM models become applicable.

## 4. Happiness from "wealth shock"

Sherman et al.’s model [13] explains how wealth shock causes negative consequences and unhappiness in literature. Like our model, they also apply EU as a measure of an individual’s subjective happiness. This is different from measuring how the sudden acquisition of wealth negatively influences the nation’s welfare. For example, the de-industrialization causes the "Dutch Disease", a severe economic recession after discovering a national gas in the 1960s. Studying individual happiness must be quantitative and therefore relies on the economics’ model such as the EU. Interestingly, [13] distinguishes the monetary and non-monetary components of individual happiness. Nevertheless, in this paper, we argue that individual happiness may come from interpersonal comparisons. More specifically, we consider subjective happiness from comparisons and distinguish between one’s own past and peers’ current situations. We next explore disappointment, including its mechanism and implications on earning and efforts. We formally answer why the sudden acquisition of wealth cannot surely bring more happiness.

In the general AM and WB model, disappointment is the unhappiness experienced if the *Frustration* caused by interpersonal comparisons exceeds the *Desirability*. In the AM model, the desirability is the marginal utility of a wage x; measured by u(S+x)-u(S). The frustration measures the negative impact of social comparisons on people’s desirability over; measured by u(S+x)-u(S+x-P). Happiness is the per-period utility level; measured by u(S+x-P)-u(S). Thus, Disappointment is another state variable and measured by -[u(S+x-P)-u(S)]. In the WB model, after the aspiration level R is entered, Disappointment is measured by -[u(S-R+x-P)-u(S)]. These state variables inherit from the general model of [2] and have been summarized in Table 2. Essentially, Disappointment is produced from a negative level of utility. People feel disappointment when their actual payoffs are lower than the compositive level of retaliation and aspiration; thereby current retaliation is a certain reference point of current payoffs.

This conceptualized disappointment in our theory is different from the literature. In a past research stream, disappointment comes from comparisons with the expectation of possible outcomes. Bell [30] assumes this expectation of being the mean, whereas Koszegi and Rabin [31] believe it is on outcomes. Masatlioglu and Raymond [32] argue that this expectation can be the past chosen lottery as the reference point. Thus uncertainties in choices can be resolved in the future. Regret is a closely related concept and is studied under the label of regret theory in the literature [33, 34]. Given a set of options, regrets come from comparisons between the option you have selected and other available options. The common part between disappointment and regret is to compare outcomes with certain reference levels.

Under the framework of WB, our analysis of disappointment begins by considering an anomaly of payoffs. How is your happiness influenced after gaining an exceptionally high payoff? Suppose that $x_{t}^{h}$ denotes an exceptionally high payoff on t received by A (she). Her peer B’ payoff *y*_*t*_ = *c* is around a normal standard of industries for all periods. The normal level of payoffs is around this standard; thus, $x_{t}^{h}\gg x_{t}\approx y_{t}$. We need to analyze the situation at periods t and t+1, respectively.

### 4.1. How $x_{t}^{h}$ influences happiness at the period t?

Generally, the payoff $x_{t}^{h}$ is supposed to generate a high level of happiness at period t. However, *surprisingly*, this payoff is very likely to produce disappointment immediately. The key is the degree of aversion to being ahead captured by the compassion factor *μ*. We first consider the normal case *x*_*t*_>*y*_*t*_ where A and B are paid with a standard level of industries. The following proposition is valid.

**Proposition** **4–1**. Given *x*_*t*_>*y*_*t*_ and *R*_*t*_ that is the A’s aspiration level at period t, concerning the individual A (she):

- When *R*_*t*_≤*y*_*t*_<*x*_*t*_, she can experience happiness.
- When *y*_*t*_<*x*_*t*_≤*R*_*t*_, she cannot experience happiness.
- When *y*_*t*_<*R*_*t*_<*x*_*t*_ is satisfied, we further consider a ratio $\bar{\mu }=(x_{t}-R_{t})/(x_{t}-y_{t})$. Then,
  (C.1) she experiences happiness if $0\leq \mu <\bar{\mu }$;
  (C.2) she experiences disappointment if $\bar{\mu }<\mu <1$;
  (C.3) she is neither happiness nor disappointment (the neutral status) if $\mu =\bar{\mu }$.

The proof of Proposition 4–1 is straightforward. In the WB model, experiencing happiness is conditioned by *xP*_*t*_>*R*_*t*_≥0. By solving the linear function *φ*(*μ*) = −*μ*(*x*_*t*_−*y*_*t*_)+*x*_*t*_−*R*_*t*_ mapping to *μ*∈[0,1), we hold the neutral status $\varphi (\bar{\mu })=0$ for $\bar{\mu }=(x_{t}-R_{t})/(x_{t}-y_{t})$. The A feels happiness if $0\leq \mu <\bar{\mu }$ and disappointment if $\bar{\mu }<\mu <1$. As the compassion factor *μ* is endogenous and fixed for A, a lower aspiration *R*_*t*_ enlarges the chances of happiness at period t. In particular, the A can surely experience happiness when *y*_*t*_≥*R*_*t*_ and surely experience disappointment when *x*_*t*_≤*R*_*t*_.

Therefore, the t-period happiness is subject to the accumulated aspiration at first and then the compassion factor. Under *y*_*t*_<*R*_*t*_<*x*_*t*_, moment utility reaches to the maximum given by HAP(t) = *u*(*S*_*t*_+*x*_*t*_−*R*_*t*_)−*u*(*S*_*t*_) when *μ* = 0. Now, we replace *x*_*t*_ by $x_{t}^{h}$ where $x_{t}^{h}\gg x_{t}\approx y_{t}$. The following proposition is valid.

**Proposition** **4–2:** **(Disappointment at the period t).** Suppose that individual A is paid by an exceptionally high payoff $x_{t}^{h}$ and compare it with the peer who is paid by *y*_*t*_, where $x_{t}^{h}\gg y_{t}$. The A will *surprisingly* experience disappointment if any of the conditions below can be satisfied:

- Her aspiration level is high such that $R_{t}>x_{t}^{h}$;
- Her compassion factor is high such that $\mu >(x_{t}^{h}-R_{t})/(x_{t}^{h}-y_{t})$.

The proof of Proposition 4–2 is straightforward. It uncovers that disappointment could derive from a significantly high and advantageous payoff in the social context. The conditions of that include (A) the high *R*_*t*_ that surpasses $x_{t}^{h}$ and (B) the high *μ* that surpasses a threshold $\bar{\mu }=(x_{t}^{h}-R_{t})/(x_{t}^{h}-y_{t})$. In sum, "abnormal" disappointment derived from the high pay is due to the intrinsic channel (i.e., the self-adaptation due to R) or the extrinsic channel (i.e., the social comparisons due to *μ*), independently or jointly.

How does the analysis of WB above vary in two degenerated models AM and AS? Under the framework of AS, compared with the standard *x*_*t*_, the $x_{t}^{h}$ surely increases DES(t) and HAP(t). Under the framework of AM, with a fixed *S*_*t*_, the t-period happiness depends on the net outcomes $xP_{t}^{h}$ where $xP_{t}^{h}=x_{t}^{h}-P_{t}$ for $P_{t}=\mu (x_{t}^{h}-y_{t})$. Experiencing happiness is conditioned by $xP_{t}^{h}>0$. By solving the linear function $xP_{t}^{h}=-\mu (x_{t}^{h}-y_{t})+x_{t}^{h}$ mapping to *μ*∈[0,1), the A surely experiences t-period happiness due to $xP_{t}^{h}>0$. More happiness comes from a smaller *μ*. If *μ* = 0, happiness is maximized as given $HAP(t)=u(S_{t}+x_{t}^{h})-u(S_{t})$. The abnormal payoff $x_{t}^{h}$ fully contributes to producing happiness. Compared with WB, happiness in AM is without any loss from social comparisons.

### 4.2. How $x_{t}^{h}$ influence happiness at the period t+1?

How an exceptionally high payoff $x_{t}^{h}$ could influence the A’s subjective evaluation over *x*_*t*+1_—a normal level of payoffs in the next period. Let us analyze from AS, AM, and then WB finally. Under AS, the payoff $x_{t}^{h}$ causes a high aspiration level as $R_{t+1}=R_{t}+\alpha (x_{t}^{h}-R_{t})$. Moment happiness is subject to the speed of establishing the aspiration. The neutral status is *x*_*t*+1_ = *R*_*t*+1_ that can be rewritten as $\bar{\alpha }=(x_{t+1}-R_{t})/(x_{t}^{h}-R_{t})$. Thus, the A experiences happiness when $\alpha <\bar{\alpha }$ and disappointment when $\alpha >\bar{\alpha }$. A larger *x*_*t*+1_ or a lower *α* tends to enlarge the moment level of happiness. Under AM, past payoffs influence current happiness through the intermediate variable *S*_*t*+1_. A larger $x_{t}^{h}$ produces a larger *S*_*t*+1_ that further mitigates the moment happiness.

In the WB model, experiencing happiness is conditioned by *x*_*t*+1_−*P*_*t*+1_>*R*_*t*+1_. Given *x*_*t*+1_≈*y*_*t*+1_, we consider *P*_*t*+1_≈0. The case thus has been degenerated as that in AS. The A will experience disappointment if $1\geq \alpha >\bar{\alpha }$. Therefore, the following proposition is valid.

**Proposition 4–3:** **(****Disappointment at the period t+1).** Suppose that the individual A is paid by an exceptionally high payoff $x_{t}^{h}$ such that $x_{t}^{h}\gg y_{t}$ and a standard level of payoff *x*_*t*+1_. The A will *surprisingly* experience disappointment at the period t+1 if the condition below can be satisfied:

$$\alpha >\left(x_{t+1}-R_{t}\right)/\left(x_{t}^{h}-R_{t}\right)$$

This proposition uncovers that an individual who receives an exceptionally high payoff in the past will surprisingly feel disappointed in the current and normal payoff, on the condition that the aspiration (retention) speed is larger than the threshold *α*.

### 4.3. Continuous disappointment across periods due to $x_{t}^{h}$

Combined with the Propositions 4–2 and 4–3, a high payoff could trigger continuous disappointment across periods. This prediction contradicts the common sense—"the more you earn, the happier you are." Under the framework of WB, the high level of payoffs indeed increases the marginal utility of this payoff immediately; yet, it meantime increases the retaliation level. When individual sensitivity of compassion is larger than the threshold $\bar{\mu }$ for $\bar{\mu }=(x_{t}^{h}-R_{t})/(x_{t}^{h}-y_{t})$, a feeling of disappointment will be suffered. In other words, the endogenous compassion (*μ*) under contain conditions may impede happiness. In the subsequent periods, on the other hand, the $x_{t}^{h}$ increases the aspiration level that goes against producing happiness. When the endogenous speed of establishing aspiration (*α*) is larger than the threshold $\bar{\alpha }$ where $\bar{\alpha }=(x_{t+1}-R_{t})/(x_{t}^{h}-R_{t})$, a feeling of disappointment will be suffered once again. In this multi-period process, the dominating effect is the extrinsic social comparisons at the period t and the intrinsic self-adaptation at the period t+1.

Therefore, more money does not necessarily buy you more happiness; it even disappoints you across periods. This prediction applies to gain a suddenly upward payoff rather than a steady high-level payoff stream. [9] sheds light on that more money cannot surely produce more happiness, which resembles our prediction here. However, it is just the opposite to ours: increasing payoffs will raise moment utility due to a low initial benchmark; the steady payoff afterward cannot increase moment utility due to the boosted benchmark. The dominating factor in their model is the transition of satiation over time. The AM model can degenerate into their satiation model if minimizing the effect of interpersonal comparison (e.g., *x*_*t*_≈*y*_*t*_ = *c* for all t). Under certain conditions, the AM thus accommodates [9]’s satiation model as a special case. The key difference is that the determinant here is the instant retaliation rather than the "quasi-satiation" benchmark—the ambition level. For example, increasing payoffs could immediately disappoint you once satisfying $\mu >\bar{\mu }$. The WB model advances AM by further incorporating the intrinsic determinant—self-adaptation, such that disappointment could be continued once further satisfying $\alpha >\bar{\alpha }$. The boosted aspiration is the dominating factor of happiness in subsequent periods.

An example makes our arguments vivid. A person who is paid by a steady payment suddenly gains a lot of money from a lottery. It could not make her happy under certain conditions (i.e., certain *R* and *μ*), for example, mental pressures derived from her social context. Subsequently, this money could further make her feel disappointed towards her normal payment because her aspiration level *R* has been boosted.

## 5. Happiness for willingness-to-work

### 5.1. Happiness for labor-supply decisions

Camerer et al. [35] study a well-known scenario on labor supply: New York cabdriver’s willingness-to-work. Workers in this type of employment have flexible self-determined work time. Their wages are generally accounted for by hours and weakly correlated between days. Similar employments include farmers, self-employed workers, or small-business proprietors. Next, we extend the analytic works toward this type of employment and exploit relations between happiness and efforts.

We first assume that people naturally tend to seek a higher level of happiness. [4] systematically distinguishes the EU from DU. DU is based on revealed preference. "The utility of outcomes refers to their weight in decisions: utility is inferred from observed choices and is in turn used to explain choices,” stated by [26]. Maximization of DU in standard choice theories is to judge the attractiveness of an option, which is deemed as absolute truth. For the EU, people do not naturally maximize happiness due to various biases in beliefs, tastes, or perceptions [36]. Under the WB model, only maximizing the left side may not be "morally wrong and self-defeating as well", because "people do try to maximize pleasure and minimize pain" ([26], p.761). It is a legitimate exercise to seek happiness or avoid disappointment by human nature in analyzing the scenario of labor supply.

Our analyses are based on a basic behavioral assumption: experiencing happiness motivates people’s willingness to work and experiencing disappointment triggers people’s unwillingness to work. With all results of Section 4 in mind, the following proposition is valid for the "cabdriver" A (she).

**Proposition 5–1.** When the A is paid an exceptionally high wage $x_{t}^{h}$ for an hour t, the WB model predicts that:

- Disappointment is surely experienced in the hour t if $R_{t}\geq x_{t}^{h}$ or $\mu >\bar{\mu }$,
  where $\bar{\mu }=(x_{t}^{h}-R_{t})/(x_{t}^{h}-y_{t})$
- Disappointment is surely experienced in the hour t+1 if $\alpha >\bar{\alpha }$
  where $\bar{\alpha }=(x_{t+1}-R_{t})/(x_{t}^{h}-R_{t})$

The proof of Proposition 5–1 is straightforward. In the hour t, a larger *R*_*t*_ tends to reduce the threshold $\bar{\mu }$, which further enhances the chance of disappointment at t. If the cabdriver decides to continue working in the next hour t+1, a larger *R*_*t*_ tends to reduce the threshold $\bar{\alpha }$, which further enhances the chance of disappointment at t+1. This is valid even if minimizing the negative effect from the social comparison at the t+1 (i.e. minimizing *P*_*t*+1_). On the contrary, if *R*_*t*_ is small enough until *R*_*t*_≤*y*_*t*_, the cabdriver can experience happiness at the hour t without considering their endogenous compassion factor *μ*.

Therefore, the WB model predicts that if a cabdriver earns an abnormal high wage at this hour, she is less likely to continue working, on the condition that her aspiration level is higher than this wage. Whether experiencing happiness from this wage predominantly depends on the endogenous characteristics of this cabdriver, which contain her compassion factor *μ* and her speed of establishing aspiration *α*.

**Proposition 5–2.** When the A is paid by $x_{t}^{h}$ and the peer is paid by *y*_*t*_ such that $x_{t}^{h}\gg y_{t}$ on t, the A is unwillingness-to-work at period t if any of the conditions below can be satisfied:

- $R_{t}\geq x_{t}^{h}$;
- $R_{t}<x_{t}^{h}$ and $\mu >\bar{\mu }$ and $\alpha >\bar{\alpha }$,

where $\bar{\mu }=(x_{t}^{h}-R_{t})/(x_{t}^{h}-y_{t})$ and $\bar{\alpha }=(x_{t+1}-R_{t})/(x_{t}^{h}-R_{t})$.

The proof of Proposition 5–2 is straightforward. The thresholds $\bar{\mu }$ and $\bar{\alpha }$ are to gangue the worker’s endogenous cognitive coefficients, *μ*, and *α*, respectively. In the next period t+1, the worker is motivated for willingness-to-work if *R*_*t*+1_ is high, especially when *R*_*t*+1_ surpasses *S*_*t*+1_.

Analyzing this within-day labor-supply scenario by the WB model, on the one hand, an exceptionally high wage in the morning may trigger disappointment immediately if *R*_*t*_ or *μ* is large enough. Cabdrivers are reluctant to continue working because they will surely experience disappointment for any wage level in the afternoon once *α* is large enough. It replicates the first part of Koszegi and Rabin’s [31] predictions and resembles [39, 40] and [35]. On the other hand, the exceptionally high wage contributes to a high aspiration level that workers use to perceive their happiness in the afternoon. The aversion to disappointment drives their willingness to work. This replicates the second part of [31]’s predictions. Once the worker quits working in the afternoon, the withdrawal of a wage perhaps triggers *craving* in the subsequent period as we explored in Section 3.

Using the WB model to analyze labor-supply decisions is based on the perception of payoffs under certain behavioral assumptions. Unlike [31] and [35], we focus on the indirect relationship between payoffs and efforts, in which the feeling of happiness (or disappointment) serves as an intermediary. The endogenous factors *μ* and α associated with personal characteristics are determinant of experiencing happiness. Note that our analysis here is in deterministic settings and riskless environments.

Next, we study interactions between happiness and incentives under the social context. We do not care how people obtain the peers’ information for comparisons interpersonally. Recently, [37] identified the conditions; thereby a company should reveal wage information to its employees, resulting in social comparisons. They found that social comparisons reduce collaborations interpersonally but could increase their willingness to ally for lowering the cost of effort. In their model, people dislike being behind more than they like being ahead. People thus are ahead-seeking and not fairness thinkers, in which cases perceived happiness could be amplified due to complacence as analyzed in [2] (Section 8.3).

### 5.2. Implications of happiness in earnings and efforts: A discussion

The classical model [35] proposes a target earnings model to explain happiness in earning and efforts. That is, a target earner will work until earnings reach a reference level and then is sharply unwilling to work. They assume an extreme version of PT’s utility function that contains a sharp kink as a reference point. When wages are less than this kink point, drivers continue to work because of a high marginal utility. Once wages reach this kink point, drivers immediately quit working because of an extremely low marginal utility. Unlike [35]’s model, our theory uses the aspiration level as the reference point. The retaliation captures people’s perception of gain or loss in a social context. Cabdrivers’ subjective happiness is thus not necessarily relative to the kink point but hinges on their ambition level accumulated in the past. Therefore, our theory implies that interpersonal comparisons also influence people’s willingness-to-works and further labor supplies.

The literature [38] empirically evidences that relative wage comparisons with sisters or sisters-in-law increase married women’s willingness to work. Farber’s reference-dependent model [39] can be interpreted as the asymmetry between gains and losses relative to a reference point caused by relative wage comparisons across individuals. Our theory uses formulated retaliation to capture psychological reactions on interpersonal comparisons and uses formulated aspiration to capture psychological reactions on self-adaptation. Only if a wage exceeds the composition of aspiration and retaliation, a cabdriver feels happy with her wage, and further motivate their willingness to work. Unlike predicted by [35] that drivers sharply quit working once the reference level is reached, happiness in our theory carries over across periods, which *indirectly* causes behaviors of unwillingness-to-work.

[31]’s model advances [35] on predicting within-day labor-supply decisions. Farber [39, 40] advocate [35] on “now earned more, later to work less”. Oettinger [41] and Fehr and Goette [42] empirically evidence the behavioral pattern of “expecting higher wage, then to work more.” [31]’s model reconciles the twofold insights: (a) exceeding a reference level tends to reduce efforts and (b) a higher expectation-based reference level tends to motivate efforts. The target wage in [35] is particularized as a notion of rational expectation that appeared in [31]. Therefore, [31] replicates [35] to indicate that the unexpectedly high wage *earlier* causes drivers’ unwillingness to work *later* for any possible wage. Besides, [31] advances this explanation as that the high expected wage motivates drivers’ willingness to work.

Our theory provides similar predictions as [31]. Assume that people are seeking happiness and averse to disappointment. The predictions of the WB model on labor supply contain two aspects: if receiving an exceptionally high wage at a period, the worker is less likely to continue work because continuous disappointment could be suffered; yet this worker is more likely to continue work because her aspiration level thus far is considerably high, especially higher than her ambition level. For another implication, the aspiration in this scenario can be interpreted as an incentive for willingness to work. A high aspiration accumulated thus far motivates people’s willingness to effort since they tend to avoid a high retraction. The retraction has been considerably large when the aspiration R is less than the ambition S since it covers the steepest utility curve in the negative zone.

## 6. Conclusion

This study explores the implications and applications of the happiness and WB model developed in [2]. We specify the utility formula as the prevailing GL utility that entails an S-shaped curve and certain loss aversion. Under this assumption, the WB model exhibits unique features and fascinating predictions in various scenarios. First, we exploit the psychological phenomenon of craving predicted by the models. In a social context, craving comes from a negative ambition. We explore its mechanisms and conditions; thereby, craving can be avoided or utilized like a double-edged sword. Second, we formally answer why more money cannot necessarily buy you more happiness. A high wage may disappoint you immediately and even disappoint you continuously across periods. Predicted by the models, the determinants are two endogenous coefficients: the compassion factor and the speed of establishing aspiration. Third, we extend related analytic works in predicting self-determined workers’ labor-supply decisions, where subjective happiness becomes the intermediary between actual wages and imminent efforts. Under certain conditions, social comparisons trigger people’s unwillingness to work, which advances classical understandings in motivations of quit-working behaviors. These findings presented in this paper uncover the substantial effect of social comparisons on individual wellbeing and many application scenarios.

In literature, [43] studied the stability of happiness and raised an interesting question: "is it possible to become a permanently happier person?" Under our theoretical framework, it could happen in theory if two conditions are held: (a) interpersonal comparisons have completely vanished, and (b) the speed of self-adaptation cannot catch up with the speed of incremental desirability. Thus, the entire analysis falls into our AS model, where an exponential smoothing of past payoffs takes effect. However, this paper aims to analyze how interpersonal comparisons influence subjective happiness in a micro level. If interpersonal comparisons are considered, maintaining a permanently higher level of happiness can be ideal. People’s moment happiness as well-formulated in our theory is dynamic, transitional, and unstable, which is subject to the current and past consumption levels of one’s own and others, one’s personality traits like easy to envy or else easy to compassion, the speed of habituating past situations, and the speed of establishing one’s ambition through past comparisons interpersonally. The innovation of our theory is exactly to incorporate and quantify all of these influence elements mentioned before; and, through a simple cardinal utility model, to measure the complex causes of happiness. More importantly, the advanced model presented in this paper perfectly predicted the psychological phenomenon of craving, explained the evolutions of happiness from the sudden acquisition of wealth, and uncovered the interactions between earnings and willingness-to-work. These implications and predictions possess both theoretical values and practical significances.

We suggest three directions for future works that respond to the limitations of this paper. First, our theory assumes people who dislike being behind more than dislike being ahead of their peers. In practice, a part of people could in turn ahead seeking and enjoy it. Whether and how the model could incorporate such complacence-driven behaviors need more exploration. Second, our theory assumes that current and past wages influence current happiness. Yet, how scheduled wages of the future could influence current happiness is still not clear. Chai’s theory [2] has shed light on that there exists bias when predicting future happiness. It is valuable to explore how to measure happiness under scheduled schemes of wages. Third, several results of this paper entail interesting hypotheses on happiness and incentives (e.g., the willingness-to-work-hard) that could be tested in laboratory or field experiments. Related empirical studies are expected in the future.
